# Supplementary material for: Effects of a Theory-Based, Multicomponent eHealth Intervention for Obesity Prevention in Young Children from Low-Income Families: A Pilot Randomized Controlled Study
Source: Nutrients. 2023 May 13;15(10):2296. doi: 10.3390/nu15102296 (PMC10222385; doi:10.3390/nu15102296)
Supplement: Supplementary file 1 [file nutrients-15-02296-s001.zip › nutrients-2373564-supplementary.pdf]

Table S1. Baseline comparison of demographic characteristics with dropouts (n = 73).

Characteristic	Completers	Dropouts	p-Value
Age (months)	26.11±8.83	28.58±6.30	0.39
Gender			0.53
Female	33 (54.1%)	8 (6.7%)	
Male	28 (45.9%)	4 (3.3%)	
Race			0.41
Biracial	22 (36.1%)	5 (41.7%)	
Hispanic	14 (22.9%)	2 (16.7%)	
Non-Hispanic White	14 (22.9%)	3 (25.0%)	
Black	11(18.0%)	1 (8.3%)	
Native Hawaiian and other Pacific Islanders	0	1 (8.3%)	
BMI-for-age z score	0.74±1.48	1.55±2.33	0.41
Weight status			0.92
Underweight	33 (54.1%)	8 (6.7%)	
Healthy weight	14 (22.9%)	2 (16.7%)	
Overweight	9 (14.8%)	1 (8.3%)	
Obesity	5 (8.2%)	1 (8.3%)	
Relationship with child			0.58
Mother	51 (83.6%)	11 (91.7%)	
Father	6 (9.8%)	0	
Foster mother	3 (4.9%)	1 (8.3%)	
Grandmother	1 (1.6%)	0	
Parental educational attainment			0.21
High school	15 (24.6%)	7 (58.3%)	
One year of college	12 (19.7%)	1 (8.3%)	
Bachelor's degree or equivalent	9 (14.8%)	0	
Some high school	8 (13.1%)	2 (16.7%)	
Two years of college	7 (11.5%)	2 (16.7%)	
Master's degree	5 (8.2%)	0	
Three years of college	5 (8.2%)	0	
Parental marital status			0.20
Never married	26 (42.7%)	10 (83.3%)	
Married	23 (37.7%)	2 (16.7%)	
Divorced	7 (11.5%)	0	
Separated	3 (4.9%)	0	
Engaged	2 (3.3%)	0	
Yearly income ($)	27,246.88± 18090.22	21,375.00± 13265.83	0.55
Household income level			0.15
Very low income	37 (60.7%)	4 (3.3%)	
Low income	9 (14.8%)	1 (8.3%)	
Non low income	4 (6.6%)	0	
Not specify	11 (18.0%)	7 (58.3%)	

Table S1. *Cont.*

Characteristic	Completers	Dropouts	*p*-Value
Parental BMI	32.18±8.56	29.37±7.00	0.33
Parental weight status			0.42
Obesity	29 (47.54)	5 (41.67)	
Overweight	22 (36.10)	3 (25.00)	
Healthy weight	10 (16.40)	4 (3.33)	

Table S2. Baseline comparison of child health behavior characteristics with dropouts (n = 73).

Variable	Completers (n = 61)	Dropouts (n = 12)	*p*-Value Between Groups
	M [SD]		
Fruit intake (servings)	0.53 [0.58]	0.46 [0.40]	0.92
Vegetable intake (serving)	0.44 [0.51]	0.48 [0.41]	0.56
Moderate-to-vigorous activity (minutes)	60.49 [32.23]	60.58 [32.31]	0.84
Sedentary behavior (minutes)	226.29 [82.43]	209.69 [75.45]	0.50
Screen viewing (minutes)	71.40 [43.38]	67.82 [34.98]	0.97

Table S3. Baseline comparison of parental psychosocial attributes and comprehensive feeding practices with dropouts. (n = 73).

Variable	Completers (n = 61)	Dropouts (n = 12)	*p*-Value Between Groups
	M [SD]		
Nutritional knowledge	11.03 [1.78]	10.83 [1.47]	0.62
Attitude	33.93 [6.19]	33.08 [6.65]	0.57
Self-efficacy	24.11 [5.16]	23.42 [5.63]	0.64
Comprehensive feeding practice	128.33 [20.39]	124.08 [11.49]	0.36

Table S4. Correlation between eHealth intervention attainment and changes in child nutritional and behavioral changes.

	Estimate	95% CI low	95% CI high	*p*-Value
Fruit intake	0.753	0.347	1.159	0.004
Vegetable intake	0.532	0.165	0.898	0.015
Screen time	27.47	10.89	43.90	0.014

Note. Results averaged mean matching values through multiple imputations. CI: Confidence Interval.
